# Precious but convenient means of prevention and treatment: physiological molecular mechanisms of interaction between exercise and motor factors and Alzheimer’s disease

**DOI:** 10.3389/fphys.2023.1193031

**Published:** 2023-06-08

**Authors:** Zikang Hao, Kerui Liu, Lu Zhou, Ping Chen

**Affiliations:** ^1^ Department of Physical Education, Laoshan Campus, Ocean University of China, Qingdao, China; ^2^ Department of Sports Medicine, Daiyue Campus, Shandong First Medical University, Tai’an, Shandong, China

**Keywords:** aging, Alzheimer’s disease, neurodegenerative diseases, exercise, nonpharmacological interventions

## Abstract

Disproportionate to the severity of Alzheimer’s disease (AD) and the huge number of patients, the exact treatment and prevention of AD is still being explored. With increasing ageing, the search for means to prevent and treat AD has become a high priority. In the search for AD, it has been suggested that exercise may be one of the more effective and less costly means of preventing and treating AD, and therefore a large part of current research is aimed at exploring the effectiveness of exercise in the prevention and treatment of AD. However, due to the complexity of the specific pathogenesis of AD, there are multiple hypotheses and potential mechanisms for exercise interventions in AD that need to be explored. This review therefore specifically summarises the hypotheses of the interaction between exercise and AD from a molecular perspective, based on the available evidence from animal models or human experiments, and explores them categorised according to the pathologies associated with AD: exercise can activate a number of signalling pathways inhibited by AD (e.g., Wnt and PI3K/Akt signalling pathways) and reactivate the effects of downstream factors regulated by these signalling pathways, thus acting to alleviate autophagic dysfunction, relieve neuroinflammation and mitigate Aβ deposition. In addition, this paper introduces a new approach to regulate the blood-brain barrier, i.e., to restore the stability of the blood-brain barrier, reduce abnormal phosphorylation of tau proteins and reduce neuronal apoptosis. In addition, this paper introduces a new concept.” Motor factors” or “Exerkines”, which act on AD through autocrine, paracrine or endocrine stimulation in response to movement. In this process, we believe there may be great potential for research in three areas: (1) the alleviation of AD through movement in the brain-gut axis (2) the prevention and treatment of AD by movement combined with polyphenols (3) the continued exploration of movement-mediated activation of the Wnt signalling pathway and AD.

## 1 Introduction

One of the direct effects of the improvement in living standards is the increase in the average age of human survival, which has increased by almost 5.6 years compared to 50 years ago. This has triggered another phenomenon, namely, the aging of the population, with an increase in the number of people older than 60 years old (Rudnicka et al., 2020). It is estimated that by 2030, 500 million people will be affected by neurodegenerative diseases, which are inextricably linked to aging, with dementia accounting for a significant proportion of the population, and Alzheimer’s disease (AD) can be observed to account for more than 65% of the population (Hou et al., 2019). AD not only affects patients’ memory and higher cognitive abilities, but is also potentially lethal, and according to a cross-sectional survey from the United States, AD is the seventh most lethal disease in the nation. In addition to the physical suffering of patients, AD plays an equally detrimental role in socioeconomic development, with global investment in AD care reaching $82.8 billion as of 2015, and estimates suggesting that this figure could rise to $2 trillion by 2030 (Gaugler et al., 2022).

AD is a neurodegenerative disease with insidious onset and progressive development, characterized by cognitive impairment, psycho-behavioral abnormalities and reduced social life functions (Dubois et al., 2021). Generally, a family tendency to develop AD is called familial AD, while a non-familial tendency is called sporadic Alzheimer’s disease (Andrews et al., 2020). The World Health Organization reports that approximately 50 million people worldwide are currently living with dementia, with Alzheimer’s disease being the most common type. Possible risk factors for Alzheimer’s disease include increased age, female gender, low education level, smoking, midlife hypertension and obesity, hearing impairment, traumatic brain injury, lack of exercise, social isolation, diabetes and depressive disorders.

The pathological changes in the brain of patients with Alzheimer’s disease are diffuse brain atrophy and are characterized by senile plaques (SP), neurofibrillary tangles (NFT) and neuronal reduction. The SP center is β-amyloid protein (Aβ), and the main component of NFT is a highly phosphorylated microtubule-associated protein, tau protein (Ju and Tam, 2022). Genetics is one of the major factors in the development of Alzheimer’s disease. Four genes have been identified to be associated with Alzheimer’s disease, namely, the amyloid precursor protein (APP) gene, the presenilin 1 (PSEN1) gene, the presenilin 2 (PSEN2) gene and the apolipoprotein E (ApoE) gene. Among them, mutations or polymorphisms in the first three genes are closely associated with early-onset familial Alzheimer’s disease, and ApoE is closely associated with sporadic Alzheimer’s disease (Riedel et al., 2016). The more recognized pathogenesis of Alzheimer’s disease suggests that imbalance of Aβ production and clearance is the initiating factor of neuronal degeneration and dementia development, which can induce a series of pathological processes such as tau protein hyperphosphorylation, inflammatory response, and neuronal death. Meanwhile, there is a wide range of neurotransmitter abnormalities in the brains of Alzheimer’s disease patients, including acetylcholine system, monoamine system, amino acids and neuropeptides (Ju and Tam, 2022).

In the clinical treatment of AD, exercise is considered to be an effective non-pharmacological treatment (Sujkowski et al., 2022), Second, prospective observational studies suggest that the benefits of regular exercise for AD are not limited to the treatment of AD, but rather to the prevention of AD, and that physical inactivity is often considered a predisposing factor for AD (Guure et al., 2017; Iso-Markku et al., 2022). Of course, many meta-analyses of randomized controlled clinical trials have also shown that exercise has a reversing effect on memory loss, decline in higher cognitive functions, AD-induced lifestyle changes, mood abnormalities, *etc.*, In AD patients (Blondell et al., 2014; Cunningham et al., 2020; Yu et al., 2020). With the advances in molecular biology and medicine, the effects of exercise on AD have been studied in more depth, starting with the possible risk factors and their effects on the course of AD, and examining the benefits of exercise on AD in detail and at a microscopic level, such as the improvement of synaptic plasticity, the reduction of neuroinflammation, and the promotion of hippocampal neurogenesis in AD patients with increased physical activity (Barnes, 2015; Luan et al., 2019).

The scope of exercise type is extremely broad, including the type of exercise, exercise intensity, exercise frequency, exercise duration, *etc.* With the addition of these factors, different types of exercise show different characteristics, and the role of AD does not seem to be the same, some researchers believe that compared to high-intensity exercise, it seems that moderate intensity sustained aerobic exercise is more suitable for AD patients (Wang S. et al., 2021). In addition, the pathways through which exercise mediates improvement in the course of AD are also being explored and discussed by researchers. The growing number of hypotheses about potential therapeutic targets for AD and the advances in medical and biological technology have opened up new possibilities to explore the potential benefits of exercise for AD.

More and more researches tend to take a molecular biology perspective to reveal how exercise works on AD through the complex and large “network” inside the human body, using the coordination of tissues, organs and systems (Lourenco et al., 2019). Among many studies, the more concentrated area is the involvement of exercise in participating in complex biochemical reactions through signalling pathways, regulating or altering major lesion components in AD, abnormal protein expression, *etc.* Interestingly, in this basis, researchers have proposed a very explored concept: the “motor factor (Lee T. H.-Y. et al., 2019)”. That is, these biochemicals are produced or expressed in response to the stimulus of exercise; in other words, although they may be produced by different tissues, the involvement or non-involvement of exercise is crucial to their action in the body.

**FIGURE 1:**
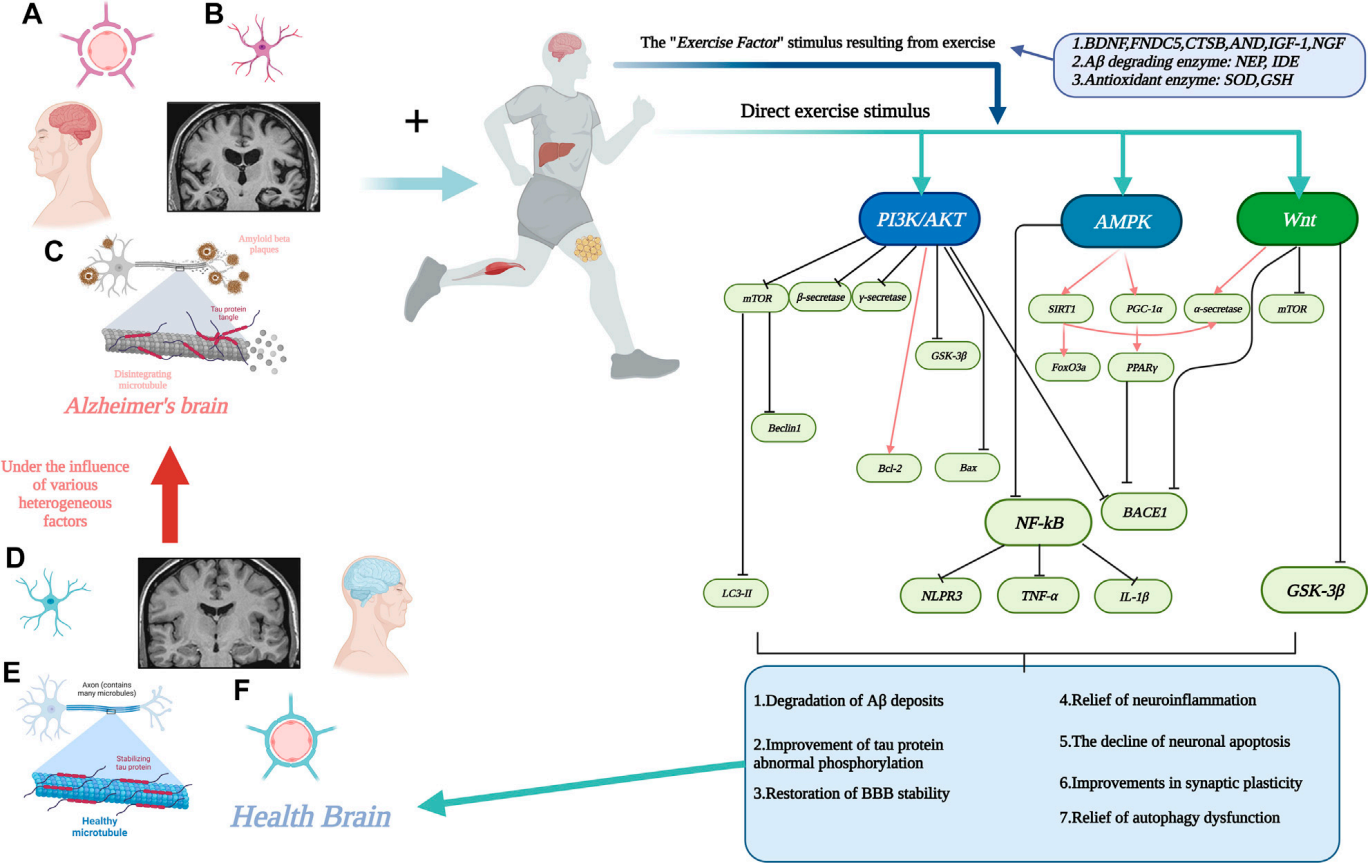
Interactions between motion and AD through signaling pathways. **(A)**. Stabilization of the blood-brain barrier disrupted by AD with increased permeability. **(B)**. Neuronal damage in the AD brain. **(C)**. Aβ deposition around neurons and neuronal fiber tangle formation due to abnormal tau protein phosphorylation in the AD brain. **(D)**. Neurons in healthy brain. **(E)**. Healthy brain without Aβ deposition and neuronal fiber tangles. **(F)**. Healthy brain with normal physiological function of the blood-brain barrier. The line segment with an arrow at the end represents facilitation and the line segment with a horizontal bar at the end represents inhibition. Created with BioRender.com.

Therefore, the aim of this review is to systematically summarize, from a molecular biology perspective, the evidence from currently available studies regarding the pathways through which exercise may be mediated (signaling pathways) and which biomarkers (involvement of motor factors) are involved in the complex chemical reactions *in vivo* to alleviate AD. Figure 1. Interactions between motion and AD through signaling pathways.

## 2 Signaling pathways, exercise and AD

**FIGURE 2:**
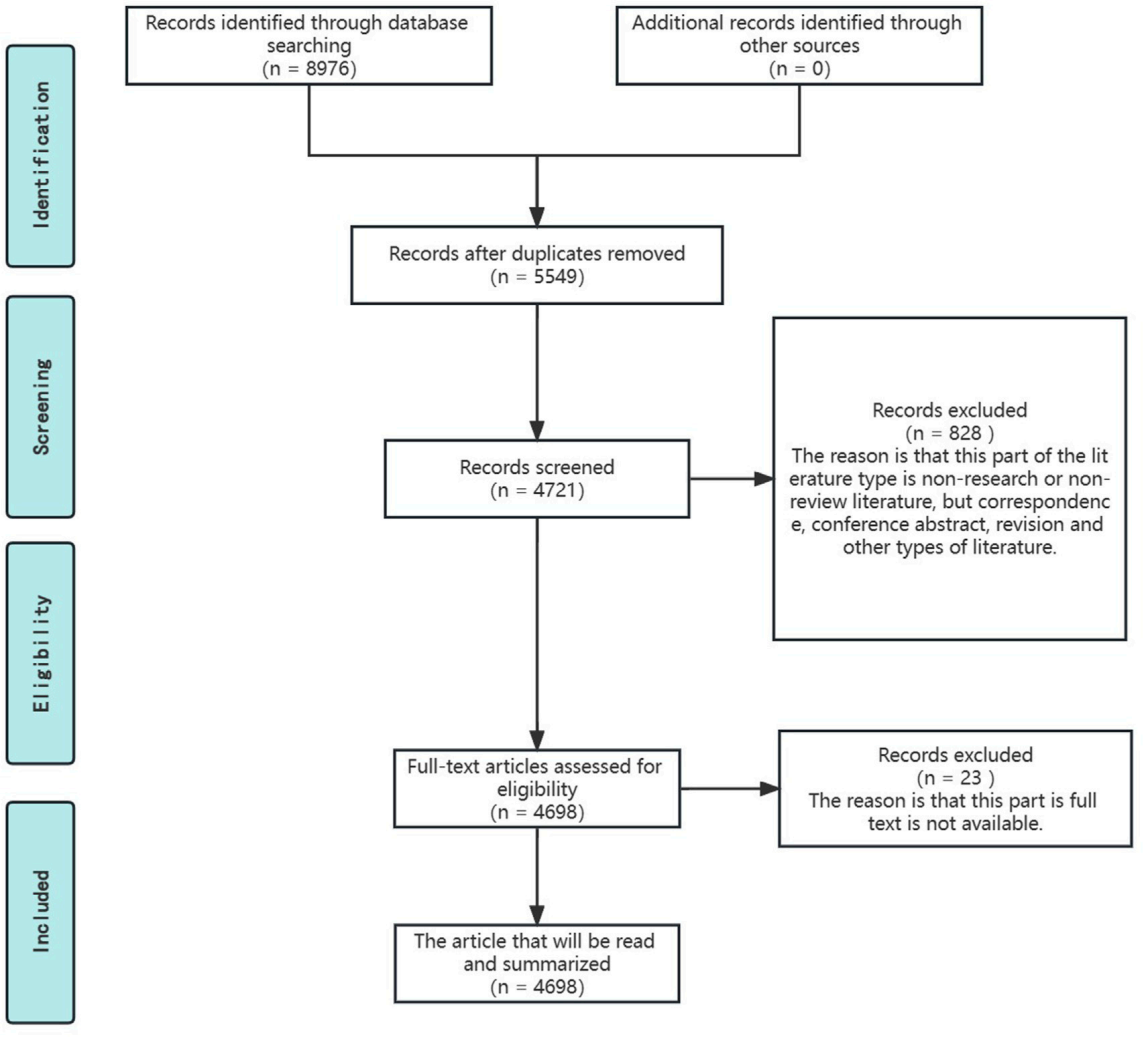
Literature reading process based on PRISMA recommendations.

Our study was based on the principles of the literature selection process suggested by PRISMA, with minor refinements, to identify the original research literature for the narrative review of this review (Page et al., 2021). The specific process is shown in the Figure 2.

### 2.1 Aβ deposition, Alzheimer’s disease and exercise

Aβ is a normal product of body metabolism, which is produced by the hydrolysis of β-amyloid precursor protein (APP) by β-secretase and γ-secretase, and under normal conditions, Aβ maintains a stable state of production and elimination in the body (Jeong et al., 2022). In contrast, increased production of Aβ and decreased clearance of Aβ in the presence of abnormal APP metabolism caused by certain reasons can lead to abnormally large deposition of Aβ, which can have toxic effects on neurons. Although the mechanism of Aβ toxicity to neuronal cells is not clear, it has been pointed out that the molecular conformation and molecular state of Aβ are related to the neurotoxicity of Aβ, and the β-sheet structure can contribute to the aggregation of Aβ into insoluble fibers, which in turn form extremely insoluble deposits and consequently generate senile plaques (SPS), leading to the development of AD. In addition, the Aβ oligomer theory suggests that soluble Aβ oligomers are more neurotoxic than insoluble fibers, causing increased apoptosis or inducing glial cells to express nitric oxide synthase (I NOS) to produce large amounts of NO to kill neurons (Cioffi et al., 2019). Based on the available evidence that Aβ deposition plays a key role in neuronal cell death and brain atrophy, which results in the onset and progression of AD, mitigating Aβ deposition is a potentially promising target for the treatment of AD (Yoon and Jo, 2012).

Exercise mitigates Aβ deposition using the Wnt signaling pathway as a mediator: In several studies, it was found that the Wnt/β-catenin signaling pathway may be associated with the dissolution of Aβ deposition in AD, and activation of Wnt/β-catenin promoted α-secretase hydrolysis of APP (Jia et al., 2019). Interestingly, inhibition of Wnt/β-catenin activity increased the level of Aβ and also increased the ratio of Aβ42/Aβ40, in addition to the finding that Wnt/β-catenin also inhibited Aβ deposition by suppressing BACE1 transcription (Parr et al., 2015).

Exercise reduces Aβ deposition mediated by PI3K/AKT: A study confirmed that in AD mice, PI3K/AKT pathway was inhibited and Aβ42 was deposited in large amounts, making the memory ability impaired, while reactivation of PI3K/AKT and inhibition of β-catabolase and γ-catabolase activities not only promoted Aβ clearance but also inhibited Aβ deposition (Um et al., 2011). As a downstream target factor of PI3K/AKT signaling pathway, mTOR has a role in negatively regulating cellular autophagy. mTOR is over-activated in AD, which inhibits cellular autophagy and leads to Aβ deposition. In some researchers, it was pointed out that exercise can reduce Aβ deposition in mouse hippocampus by regulating PI3K/AKT signaling pathway. After 12 weeks of running-table exercise, the expression levels of phosphorylated PI3K (p-PI3K) and phosphorylated AKT (p-AKT) in the hippocampus of AD mice were found to be significantly increased, while the expression levels of mTOR returned to normal and the abnormal cellular autophagy was alleviated, while the expression of Beclin1 and LC3-II, which are associated with reduced Aβ deposition, increased and the Aβ content decreased, thus suggesting that the alleviating effect of exercise on AD may be achieved by activating the PI3K/AKT signaling pathway to alleviate the abnormal cellular autophagy in AD (Kang and Cho, 2015). As mentioned previously, it is possible that exercise may also act on SPS as an AD causative factor through the PI3K/AKT signaling pathway, and the possible mechanism is that exercise inhibits BACE1 expression, thereby suppressing Aβ production and reducing Aβ deposition.

Exercise alleviates Aβ deposition through the AMPK signaling pathway: AMPK is present in all mammalian cells as a fuel-sensing enzyme, and numerous studies have confirmed that an increase in AMP/ATP ratio leads to AMPK activation in skeletal muscle, while exercise is also considered to be the most significant physiological activator of AMPK (Jeon, 2016). Physical exercise is often considered a cheap and useful intervention for the treatment of some diseases, and its effects are also attributed to the activation of AMPK (Kjobsted et al., 2018). It is well known that PGC-1α and SIRT1 are downstream factors of AMPK and their expression is positively correlated with AMPK activation, while in AD, PGC-1α and SIRT1 are often considered as important targets for Aβ alleviation. After 35 sessions of moderate treadmill exercise, it was found that learning and memory deficits were improved in AD mice, and a reduction in Aβ deposition was observed. It is hypothesized that the possible mechanism is due to the activation of AMPK by long-term exercise which in turn upregulates the expression of PGC-1α, and the PPARγ activity regulated by it is similarly increased, both of which bind to the promoter of BACE1 and inhibit the transcription of BACE1, thus reducing Aβ42 and Aβ40 production, reducing Aβ deposition (Koo et al., 2017). A recent study suggested that after prolonged exercise activation of AMPK increases the expression of SIRT1, which is regulated by it, and subsequently shifts APP to the α-transferase pathway. Possible mechanisms include upregulation of SIRT1 to activate RARβ to further activate ADAM10, and in addition SIRT1 can deacetylate FoxO transcription factor 3a (FoxO3a) in the nucleus, causing ROCK1 is often phosphorylated at the Ser655 site of APP, and inhibition of its activity can reduce the β-catabolic pathway of APP and shift APP to the α-hydrolase pathway, thereby increasing the content of αAPPs and reducing Aβ deposition (Bedada et al., 2021).

It has also been suggested that Aβ deposition, in addition to the overexpression of BACE1, is associated with the ubiquitin-protease system (UPS), an enzyme system whose main function is to degrade abnormal intracellular or extracellular proteins, which is involved in restoring protein homeostasis and maintaining normal cellular function (Al Mamun et al., 2020a). In AD, a downregulation of UCHL-1 expression is often observed, while interestingly, in another study, UCHL-1 was found to be negatively correlated with BACE1. The possible mechanism is thought to be that the expression of UCHL-1, which is an E3 ligase, affects the function of UPS and thus the clearance of Aβ deposition. In contrast, exercise activates PI3K/AKT, which in turn upregulates the expression of UCHL-1 after activation, thereby improving Aβ clearance (Xu et al., 2022).

### 2.2 Abnormal phosphorylation of tau proteins, Alzheimer’s disease and exercise

Tau protein, a highly soluble microtubule-associated protein (MAP), is abundant in neurons of the central nervous system (CNS). As a member of the MAPs family of proteins, tau protein mainly acts on the distal axon to maintain microtubule stability and flexibility, while other proteins such as MAP6 protein mainly acts on the proximal axon to fix microtubules, and MAP2 mainly maintains dendritic stability (Martin et al., 2013). Tau proteins interact with microtubule proteins (Tubulin) to stabilize microtubules while driving Tubulin assembly within microtubules. Tau proteins control microtubule stability through heterodimerization and phosphorylation (Ye et al., 2022). The phosphorylation of tau is regulated by a dynamic balance between tau kinase and phosphatase activities. Disruption of this balance is an important cause of abnormal tau phosphorylation, which causes tau protein aggregation and constitutes neuronal intracellular aggregates, thus causing neuronal fiber tangles (Al Mamun et al., 2020b).

The protein kinase of Tau, mainly PDPK, causes phosphorylation of tau through three pathways: A. Overexpression of GSK-3β induces abnormal phosphorylation of tau leading to neurodegeneration, resulting in a series of pathologies such as neuronal apoptosis, hippocampal neurodegeneration, memory deficits, increased neuroinflammatory Aβ deposition and reduced acetylcholine synthesis (Llorens-Martin et al., 2014). B. The most active form of CDK5, CSK5/p25 polymer, induces increased tau phosphorylation and neurodegeneration and induces neuronal apoptosis (Engmann and Giese, 2009). C. MAPK signaling pathway controlled by p38, Erk1/2, JNK1/2/3, where JNK activation is also involved in increased γ-secretase activity associated with increased production of Aβ (Ploia et al., 2011).

The activity of the Wnt signaling pathway is thought to be associated with hyperphosphorylation of tau in AD. In mice, dysfunction of Wnt was found to lead to phosphorylation of tau proteins, Thr231, Ser235 and AT8, resulting in hyperphosphorylation of tau, and reactivation of the Wnt signaling pathway revealed that hyperphosphorylation of tau was regulated, and in addition to this, GSK-3β activity was subsequently inhibited. It is hypothesized that in AD, inhibition of the Wnt signaling pathway leads to overexpression of GSK-3β, which induces hyperphosphorylation of tau and leads to neurogenic fiber tangles. In contrast, 12 weeks of aerobic exercise on a running platform upregulated Wnt-3 expression in the hippocampal tissue of MCI mice and inhibited GSK-3β expression, which somewhat alleviated Tau hyperphosphorylation and improved cognitive performance in mice.

As mentioned previously, a useful target for improving tau hyperphosphorylation and NFT deposition is thought to be GSK-3β. In addition to the Wnt pathway, the PI3K/AKT signaling pathway is also thought to be critical for reducing GSK-3β activity in AD. Exercise has been shown to have an effect on the activation of the PI3K/AKT signaling pathway, and exercise is beneficial in increasing the content and activity of PI3K in the hippocampus of AD brain, prompting the activation of Akt at Thr308 and Ser473, and the activated Akt inhibits GSK-3β activity by inhibiting GSK-3β phosphorylation at the Ser9 site, thus achieving the inhibition of abnormal Tau phosphorylation and improve NFT (Fang et al., 2018a). In addition, another study found that a single acute exercise session could also have the same effect, but with a time-limited effect, i.e., GSK-3β activity decreased immediately after exercise and then recovered (MacPherson et al., 2015), However, the effects of acute exercise on AD has remained controversial, with some suggesting that the oxidative stress that may be induced after acute exercise may be very detrimental to AD (Jin et al., 2015).

**FIGURE 3:**
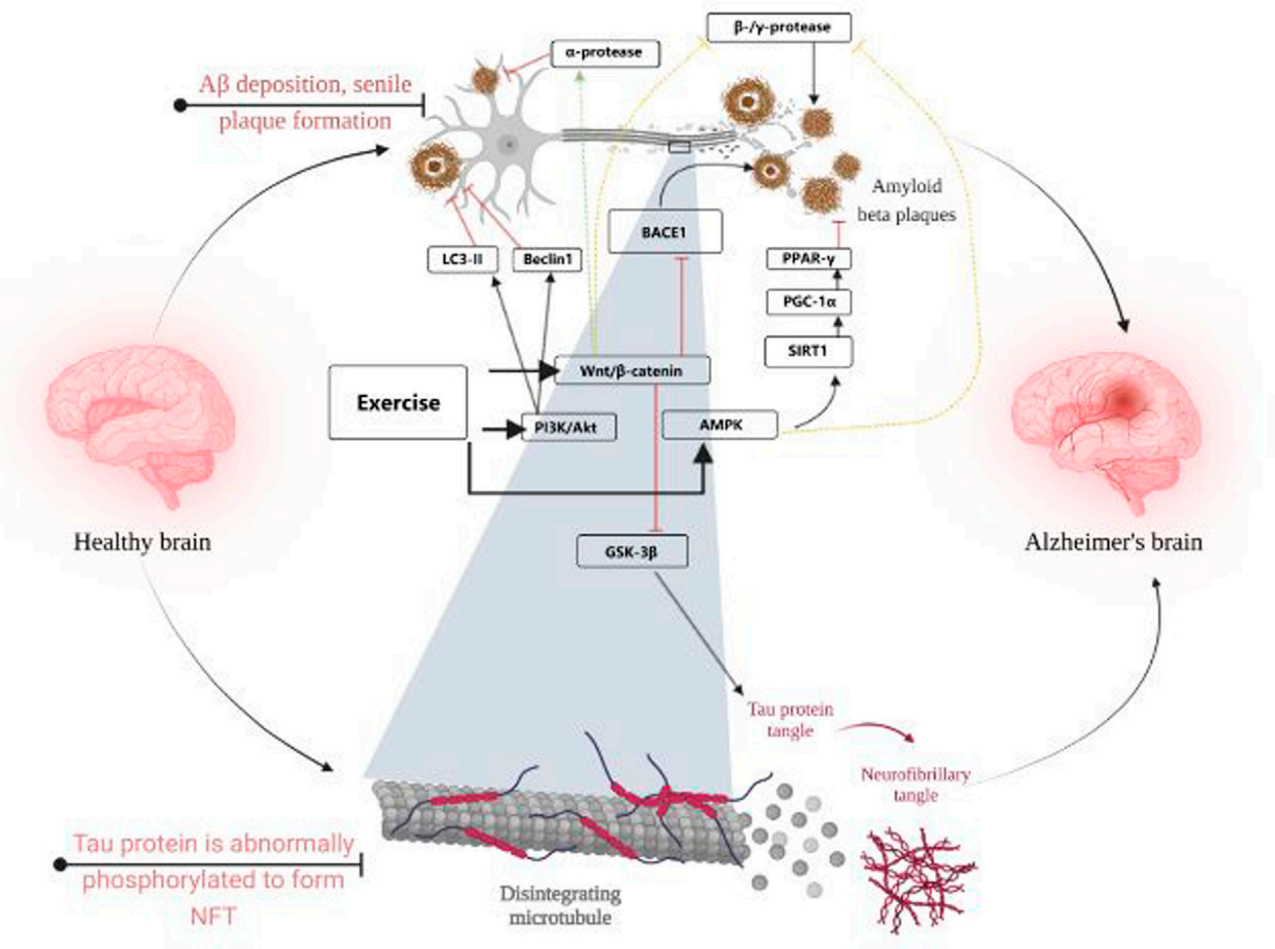
Visualises the content of 3.1 and 3.2. Created with BioRender.com.

The AMPK signaling pathway is also often thought to be closely associated with AD. In this study, it was found that p-AMPK levels were positively correlated with p-AKT levels, and after prolonged exercise, p-AMPK levels were significantly elevated and phosphorylation of tau proteins at the Ser396 and Ser262 sites was inhibited. Similarly, mTORC1/P-70-S6K, which is regulated with AKT, was also activated and served to inhibit tau protein phosphorylation at the Ser396 site (Joa et al., 2017). In addition to this, some researchers have suggested that acetylation levels of tau are often associated with promoting tau hyperphosphorylation, and that aerobic exercise can reduce Ac-tau levels (Mankhong et al., 2020), sirt1, which is associated with changes in Ac-tau levels, was not found to be associated with exercise, i.e., it is inconclusive whether exercise can upregulate sirt1 and thus reduce Ac-tau levels. However, it is well established that increasing sirt1 levels can improve tau hyperphosphorylation in AD (Chen et al., 2022). Sports are widely considered to be sirt1 activators (Vargas-Ortiz et al., 2019), The aberrant phosphorylation of tau protein by exercise may be achieved through sirt1. Figure 3 visualises the content of 3.1 and 3.2.

### 2.3 Autophagic dysfunction, Alzheimer’s disease and exercise

Autophagy is an evolutionarily conserved and important process for the turnover of intracellular material in eukaryotes. In this process, some damaged proteins or organelles are wrapped in autophagic vesicles with a double membrane structure and sent to lysosomes for degradation and recycling to maintain cellular metabolic homeostasis and self-renewal (Nixon, 2013). In normal neurons, autophagic activity is active and efficient, but in AD, autophagy is impaired, and it has been suggested that dysfunction of autophagy and lysosomes precedes lesions in AD, and that this impairment has a facilitating effect on neuronal apoptosis and plaque formation (Zhang et al., 2022). Normal autophagy can help clear Aβ deposition and abnormally phosphorylated tau proteins. Downregulation of Beclin1 and LC3-II/I ratios was observed in AD mice (Lucin et al., 2013) and mTOR expression was upregulated (Liu et al., 2021), suggesting that normal autophagic activity is inhibited.

In their study, Liu et al. observed the activation of the AMPK/Beclin1 signaling pathway by aerobic exercise, thus serving to regulate abnormal autophagy in AD (Liu W. et al., 2019). Their further study found that aerobic exercise may activate the APN-AdipoR1/AMPK signaling pathway in mice to achieve the effect of regulating autophagy-lysosome pathway abnormalities in AD, thereby alleviating AD lesions. Adiponectin (APN) is often thought to have neuroprotective effects and is useful in alleviating cognitive impairment and memory loss (Jian et al., 2022). It is likely that exercise acts through APN and its receptor AdipoR1 for the recovery of neurodegenerative diseases.

The mTOR is an important regulator for autophagy, and overexpression of mTOR induces abnormal autophagic function. One of the upstream signaling pathways of mTOR is PI3K/AKT. In AD, it was found that inhibition of PI3K/AKT and upregulation of mTOR expression affect autophagy (Heras-Sandoval et al., 2014). According to Li et al., PI3K/AKT was activated in mice after high intensity interval training and medium intensity continuous training interventions, which inhibited mTOR expression and was helpful for the restoration of autophagic function. In addition to this, the expression of autophagy protein Beclin1 was also found to be upregulated after 12 weeks of running table exercise, which increased the autophagic activity that was also altered due to AD (Kang and Cho, 2015). Therefore, it is believed that the amelioration of abnormal autophagy function in AD by exercise could also exert its effect through the PI3K/AKT signaling pathway.

### 2.4 Synaptic plasticity, Alzheimer’s disease and exercise

In AD, the neurotoxic effects of Aβ can affect the structural part of the postsynapse, particularly important for PSD-95, leading to inhibition of LTP (a major form of synaptic plasticity reflecting changes in physiological activities such as neuroelectricity in synaptic plasticity) and synaptic dysfunction, causing related changes in learning and memory abilities (Tu et al., 2014). The protein expression level of PSD-95 is also often used as a practical indicator of synaptic plasticity in the mouse hippocampus (Shao et al., 2011).

Also, Wnt activation can upregulate PSD-95 and then LTP, thus causing enhanced learning and memory capacity (Oliva et al., 2013). Ren et al. found that prolonged physical activity significantly inhibited DKK1, an inhibitor of Wnt, compared to sedentary patients, resulting in a decrease in DKK1 levels in patients (Ren et al., 2017). In contrast, overexpression of DKK1 significantly inhibits Wnt activity, which could explain the much higher levels of DKK1 found in the hippocampus of a subset of AD patients at autopsy and their synaptic degeneration (Ren et al., 2019). The mechanism by which long-term exercise improves synaptic plasticity via Wnt may be that exercise downregulates the abnormal upregulation of DKK1 expression in AD, reversing to some extent the abnormalities of Wnt and its regulated PSD-95 and LTP due to DKK1 overexpression.

### 2.5 Neuronal apoptosis, Alzheimer’s disease and exercise

In AD, in addition to the aforementioned changes in synaptic plasticity, synaptic damage is often observed, and this also causes neuronal loss (Fricker et al., 2018). As a key pathway controlling cell survival, many studies have found that the absence or inhibition of Wnt makes neurons more susceptible to apoptosis induced by Aβ deposition (De Ferrari and Moon, 2006; Rosso and Inestrosa, 2013; Pascual-Vargas and Salinas, 2021). Also, in AD, finding ways to reactivate Wnt could rescue this detrimental effect. Despite controversy, new evidence suggests that hippocampal neurogenesis in humans does exist in the aging brain and realizes a dramatic decline in the AD brain. The likely mechanism is the downregulation of Wnt proteins and messy proteins, while DKK1 expression is upregulated, combined with reduced levels of Wnt protein astrocytes due to aging, thereby disrupting adult hippocampal neurogenesis.

**FIGURE 4:**
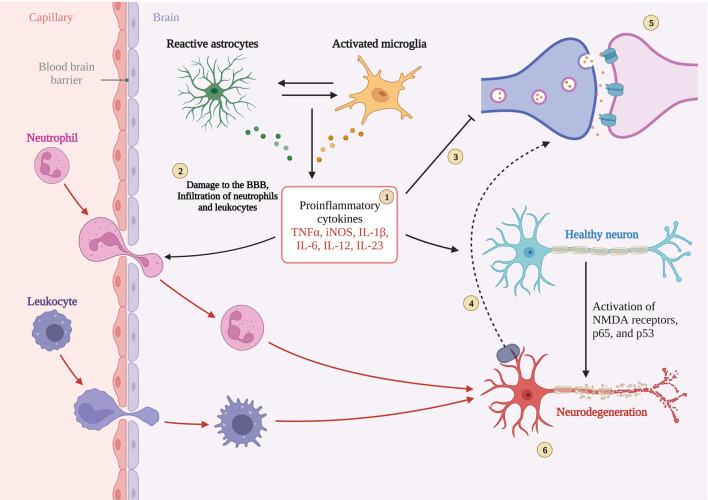
Interaction between synaptic function, blood-brain barrier, neuroinflammation in AD. 1. Sustained activation of microglia and astrocytes leading to cytokine overexpression. 2. Destabilization of the BBB leading to peripheral cytokines into the CNS.3 and 5 Inhibition and disruption of synaptic function by excess cytokines.6. Neuronal damage and death caused by cytokines.4. Magnified illustration of neuronal terminals. Created with BioRender.com.

In AD, it is often accompanied by apoptosis of neuronal cells, and in the elderly, neuronal survival is important for the health of the organism. Among all apoptotic pathways, the Bcl-2 protein family is the downstream protein family of all pathways and is essential for apoptosis, especially Bcl-2, which plays an anti-apoptotic role, and Bax, which plays an apoptotic role. In normal brain, Bcl-2 is highly expressed and saves cells from apoptosis, while in AD brain, Bcl-2 expression is suppressed, Bax is overexpressed, and Bcl-2/Bax homeostasis is disrupted, triggering neuronal apoptosis. In the latest study, it was found that aerobic exercise improved neuronal apoptosis and was accompanied by activation of PI3K/AKT Further study revealed that it was the activation of PI3K/AKT that reversed the decrease of Bcl-2 expression and inhibited Bax expression, allowing the balance to be restored (Peng et al., 2022). Figure 4. Interaction between synaptic function, blood-brain barrier, neuroinflammation in AD.

### 2.6 Neuroinflammation, Alzheimer’s disease and exercise

In AD, although the exact mechanism of its pathogenesis is unknown, the more accepted hypotheses are the Aβ hypothesis and the NFT hypothesis due to Tau. In addition, more than 30 years ago, some researchers suggested that AD may also be associated with neuroinflammation, but there is no definite conclusion to prove whether neuroinflammation is the cause or effect of AD (Calsolaro and Edison, 2016). Under normal circumstances, moderate inflammation produced by the organism will subside on its own, but prolonged inflammation can lead to the development of chronic inflammation, i.e., the organism does not recover properly. Neuroinflammation, on the other hand, refers to the inflammatory response of the CNS with neuronal damage (Thawkar and Kaur, 2019). There is abundant evidence that such neuroinflammation is often observed in AD, in addition to the activation of astrocytes and microglia associated (Lyman et al., 2014; Calsolaro and Edison, 2016; Guo S. et al., 2022). These two types of cells are essential for the inflammatory response of the CNS. It is generally accepted that microglia are in a “quiescent” state under normal conditions, but can be in a state of monitoring of the brain and its environment, and are often considered to be neuroprotective cells with phagocytic functions and release of neurotrophic factors. Under abnormal conditions, microglia are activated, and although they do initially have a defensive role, if abnormal stimuli persist, chronic neuroinflammation occurs, and the release of cytokines (TNF-α, IL-1β, *etc.*) leads to a proinflammatory response, and the persistence of these cytokines continues to induce the production of APP in the AD brain, which itself has a disrupted Aβ homeostasis and produces more APP. Also, the AD brain, whose Aβ homeostasis is disrupted, produces more Aβ due to the increase of APP, forming a vicious circle (Kwon and Koh, 2020). In addition, Aβ can stimulate the NF-kB signaling pathway, which is a downstream target of pro-inflammatory cytokines (Sivandzade et al., 2019). Activated microglia also release substances such as free radicals, reactive oxygen species and neurotoxic substances that are detrimental to neuronal survival and normal synaptic function. The effects of neuroinflammation on the course of AD are not only accomplished by exacerbating Aβ deposition and NFT, but are also mixed with more complex factors. Therefore, the suppression of neuroinflammation may be a potential way to treat AD.

NF-kB, as an important pro-inflammatory signaling pathway in AD neuroinflammation, is involved in the expression of almost all inflammatory cytokines in AD. NF-kB is nucleated and overexpresses pro-inflammatory factors such as IL-1β and TNF-α to trigger neuroinflammation, while IL-1β and TNF-α can in turn act as agonists of NF-kB in reverse (Zhao et al., 2019). The activation of PI3K/AKT signaling pathway will play a role in inhibiting the activation of NF-kB. After 8 weeks of swimming training, the expression of p-PI3K and p-AKT was upregulated in the hippocampus of rats, the activity of NF-kB was inhibited, and the expression of TNF-α, NO, IL-1β and other pro-inflammatory factors regulated by them were downregulated (Wang et al., 2019).

**FIGURE 5:**
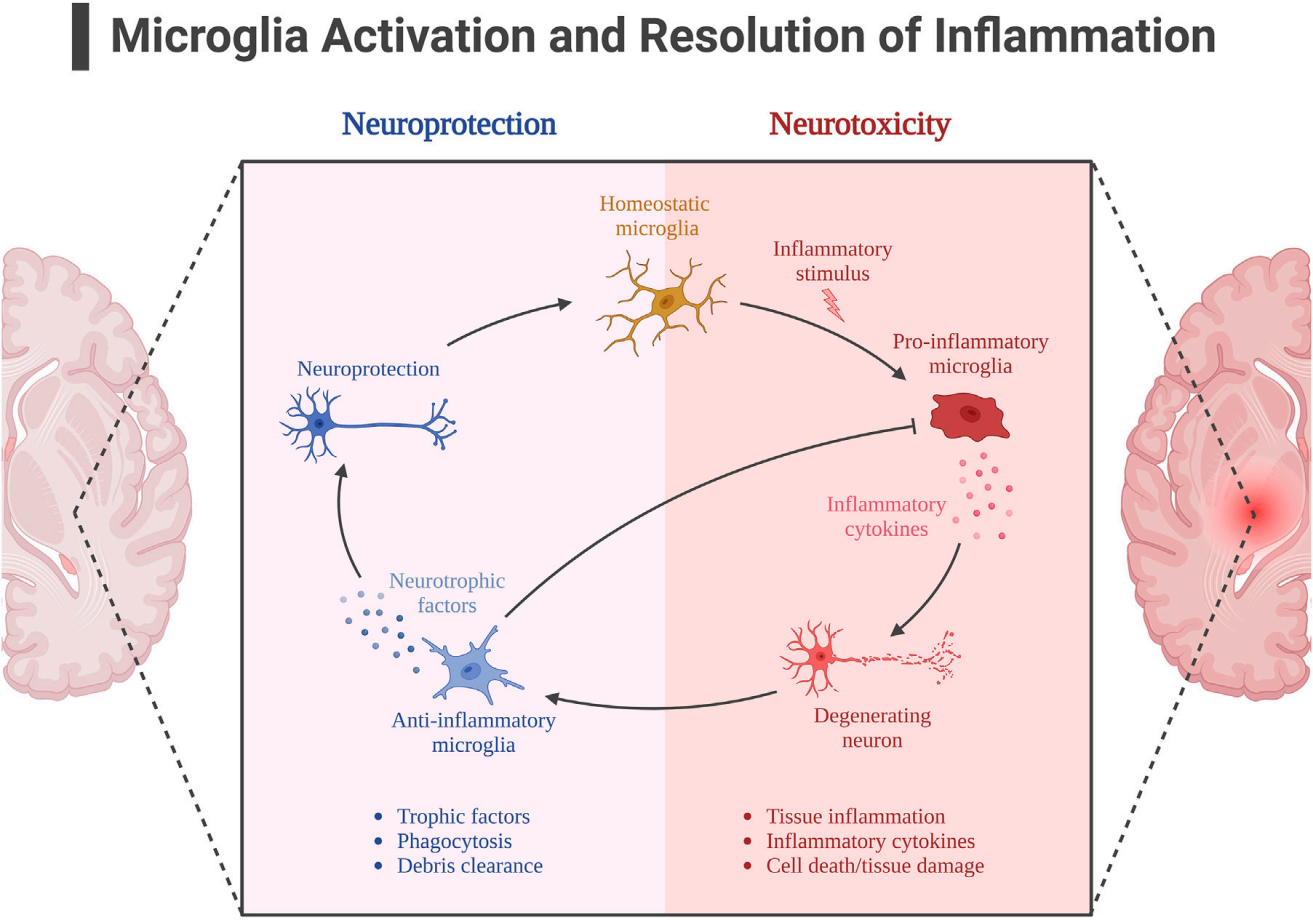
Neuroinflammation with microglial activation in AD. Created with BioRender.com.

The activation of AMPK signaling pathway by exercise is well documented, and AMPK is also considered as a “star signaling pathway” to improve some diseases, and some of the beneficial effects of AMPK are achieved through its regulation of downstream targets, such as SIRT1 and PGC-1α. Although SIRT1 has been shown in many studies to be useful in alleviating neuroinflammation in AD, it is unfortunately unknown whether the alleviating effect of exercise on neuroinflammation is mediated through the AMPK/SIRT1 axis, as SIRT1 can be activated by more than just AMPK. Exercise is important for SIRT1 expression, and in neuroinflammation in AD, SIRT1 directly deacetylates NF-kB at the p65 site, resulting in inhibition of NF-kB overactivation (Koo et al., 2017). In addition to IL-1β, TNF-α, pro-inflammatory factors that have been shown to be directly regulated by NF-kB, there is a pro-inflammatory factor NLRP3 that is also regulated by SIRT1. It is known that NLRP3 expression must be controlled by NF-kB, and overexpression of NLRP3 is often observed in insulin-resistant mice. Thus exercise appears to alleviate neuroinflammation through the SIRT1/NF-KB/NLPR3 axis (Zhao et al., 2023). According to previously described, microglia activation is also associated with NF-kB mediation, and protein expression of SIRT1 increases after exercise, thereby activating Nrf2 and PGC-1α to inhibit microglia activation and alleviate Aβ deposition and NFT formation (Fang et al., 2018b; Wu et al., 2018). Figure 5. Neuroinflammation with microglial activation in AD.

### 2.7 Blood-brain barrier, Alzheimer’s disease and exercise

Although controversial, recent studies suggest that the blood-brain barrier (BBB) is inextricably linked to AD, a functional unit that divides brain tissue from the peripheral environment, consisting of vascular endothelial cells tightly connected to each other by tight junction proteins (TJs) and adhesion-linked proteins, and pericytes and astrocytes covering the endothelial cells (Sweeney et al., 2019). It plays a role in limiting the entry of peripheral macromolecular proteins, cytotoxic substances, and peripheral inflammatory factors into the CNS, and maintaining the stability of the CNS internal environment and normal brain cell function (Liu X. et al., 2019).

Impairment of normal physiological function of BBB is often found in AD brain, such as crosstalk between neuroinflammation and reduced BBB stability, which is maintained in normal state, whereas in AD brain, the increased permeability of BBB allows the opportunity for peripheral inflammatory factors to enter the brain, which in turn is overexpressed by AD, possibly via NF-kB pathway (Vest and Wyss-Coray, 2022). This part of inflammatory factors enters the CNS, which activates glial cells and induces the differentiation of M1 phenotype, increasing the inflammatory state in AD brain, which in turn continues to impair the normal physiological function of the BBB and causes endothelial cell damage, forming a cycle. In addition, abnormal BBB function is often thought to be involved in the process of Aβ deposition. In normal brain, Aβ protein is transported from the brain to the brain via the BBB, whereas abnormal BBB function may lead to impairment of this process and abnormal clearance of Aβ transport thus causing Aβ deposition (Nelson et al., 2016; Montagne et al., 2017; Profaci et al., 2020).

In some studies on the alleviation of the AD process through physical activity it was found that exercise might possibly reverse the disruption of the BBB stability in the AD brain.

As previously mentioned, TJs are molecular substances that act as anchors to produce the BBB, and some proteins expressed by TJs are important for maintaining BBB stability, such as the claudin family and occludin. Downregulation of claudin-3 and -5 expression was observed in AD brain, while a protein that inhibits tight junctions in the brain, endothelial cell adhesion molecule 1 (PECAM -1) was upregulated. In contrast, regular physical exercise restored the expression of claudin-4 and occludin to basal levels in the brains of AD mice, while downregulating the expression of PECAM-1 for maintaining tight links in TJs (Malkiewicz et al., 2019). Interestingly, overexpression of GSK-3β may shorten the half-life and suppress the expression levels of ocludin and claudin-5, while physical exercise in turn suppresses the expression levels of GSK-3β in AD brains and indirectly restores TJs. In addition, in a study of AD induction by ALCL3 injection, it was found that physical exercise not only promoted ocludin and claudin-3 expression increases, but also decreases the downregulation of inflammation-related PHF-3 and TNF-α expression (chun et al., 2022).

**FIGURE 6:**
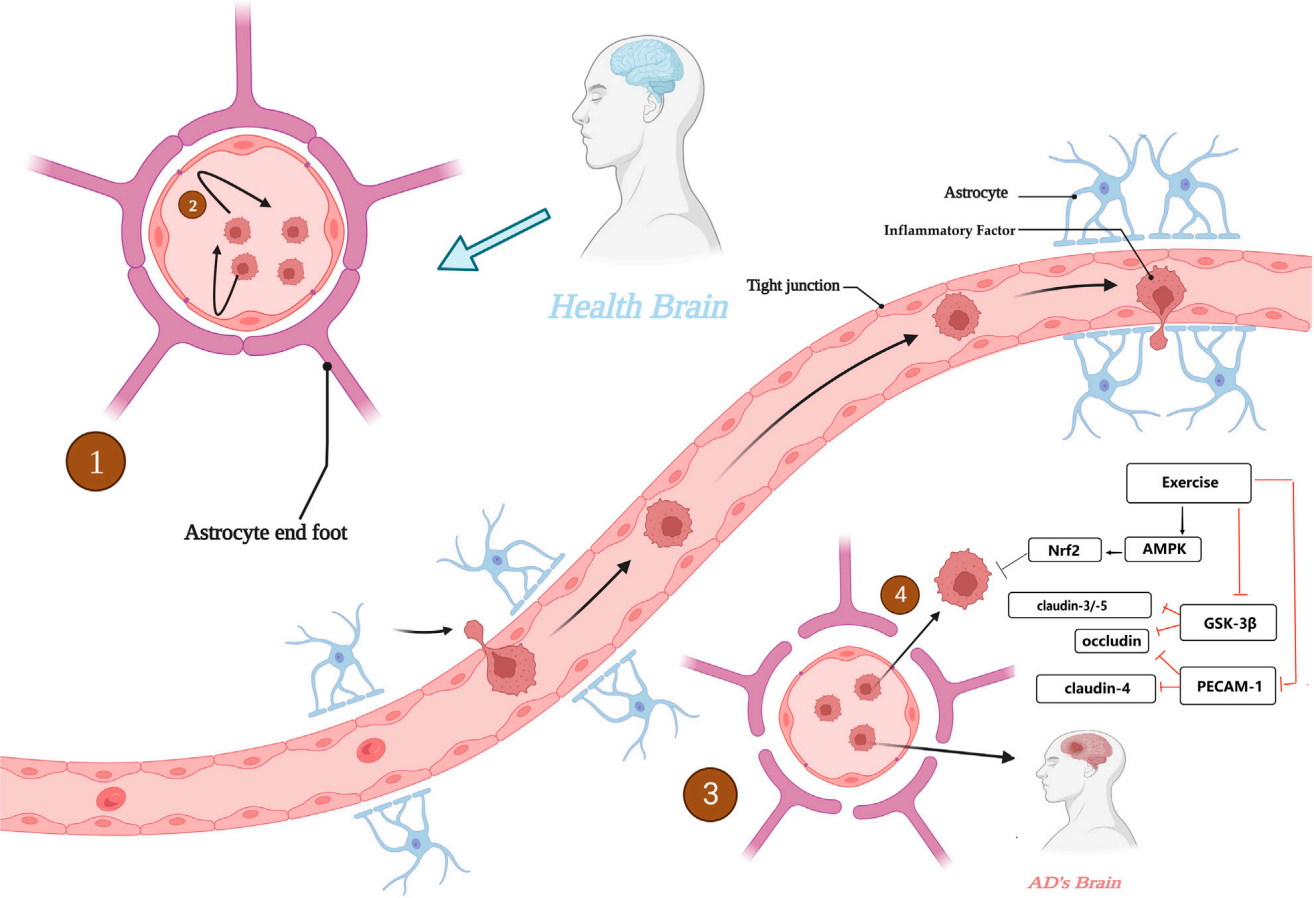
Alterations in blood-brain barrier function in AD. 1. BBB with normal physiological function in healthy brain. 2. BBB with normal physiological function to maintain the CNS from interference by peripheral harmful factors. 3. BBB with destabilization and increased permeability in AD brain. 4. The BBB, which is unable to function as a barrier, invades the CNS. Created with BioRender.com.

It is the anti-inflammatory effect of exercise that may have a “blocking” effect on the vicious cycle between CNS neuroinflammation and BBB. For example, the secretion of the anti-inflammatory factors IL-6 and IL-10 is increased and the secretion of IL-1β and TNF-α is decreased. In addition, oxidative stress is often thought to be involved in the crosstalk between neuroinflammation and the BBB, with oxidative stress states causing disruption of the tight linkage state of TJs, while physical exercise can exert antioxidant effects via the AMPK/Nrf2 pathway to maintain TJs and protect neurons (Małkiewicz et al., 2019). It is also noteworthy that disruption of the BBB due to chronic inflammation has been observed not only in AD patients but also in patients with T2DM, dyslipidemia, obesity and insulin resistance, which are often associated with chronic inflammation and are often thought to be associated with an increased risk of AD. The role of exercise in AD risk prevention by eliminating the crosstalk between BBB and chronic inflammation cannot be ignored (Bertram et al., 2016; Brown et al., 2022). Figure 6. Alterations in blood-brain barrier function in AD.

In conclusion, BBB plays an important role in alleviating the course of AD and preventing the risk of AD, and maintaining the stability of BBB may be a new target for the prevention and alleviation of AD, is a means that cannot be ignored

**FIGURE 7:**
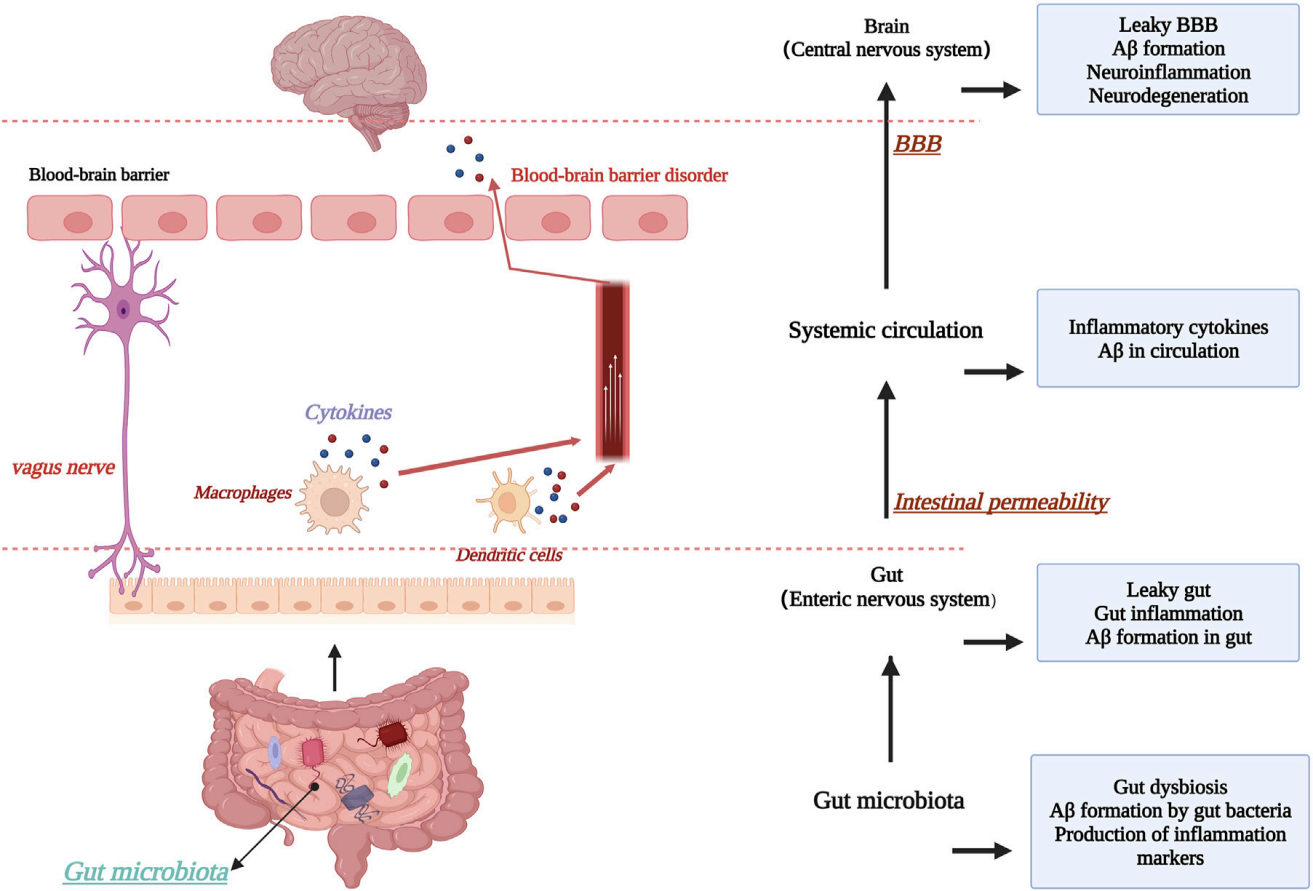
Schematic diagram of the relationship between the “brain-gut axis” and the blood-brain barrier. Created with BioRender.com.

The blood-brain barrier is of great interest to researchers as an important defense mechanism in the human body. Based on this, it has been suggested that microorganisms in the gut and intestinal tract may interconnect with the CNS through the “brain-gut axis”, i.e., forming a bidirectional “microbe-gut-brain” pathway, in which the blood-brain barrier plays an important role. In AD brain, an increase in the abundance of harmful intestinal microorganisms was found (Goyal et al., 2021), and consequently, the type of short-chain fatty acids (SCFAs) produced by the intestinal microbiota changed, which is an important nutrient for maintaining the stability of the intestinal barrier and the BBB, and significant changes in SCFAs adversely affected the maintenance of BBB stability (Wang D. et al., 2021). For example, in mice treated with antibiotics, changes in intestinal flora species were found, with the consequent effect of suppressing SCFA homeostasis and expression levels of the claudin family of proteins and occludin proteins, two transmembrane proteins that are important for the maintenance of TJs, as discussed earlier (Wang, 2023). This highlights the relationship between maintaining a stable gut microbiota and BBB stability. In addition, chronic high-sugar and high-fat diets can also disrupt the normal gut microbiota, thereby inhibiting tight junction proteins. Thus, the gut microbiota may be an important target for improving AD and maintaining BBB stability, but current studies are controversial and therefore further research is needed to determine this (Bou Zerdan et al., 2022). Figure 7. Schematic diagram of the relationship between the “brain-gut axis” and the blood-brain barrier.

## 3 Exerkines, Alzheimer's disease and exercise

There is a consensus that physical activity has beneficial effects on the body. The first half of this paper discusses the benefits of physical activity on AD in the context of signaling pathways, starting from AD and its possible causative factor. In addition, recent studies have focused on biomolecular substances called " Exerkines”, which are so named because they almost always act on the extracellular environment through autocrine, paracrine or endocrine action in response to exercise (Chow et al., 2022). Exerkines exert beneficial protective effects on CNS by crosstalking systems, tissues and organs, including but not limited to promoting neuronal regeneration, improving synaptic plasticity, and alleviating memory and higher cognitive decline.

### 3.1 Nerve-exercise-exerkines

BDNF is found in a wide range of areas such as the CNS, peripheral nervous system, endocrine system, bone and cartilage tissues, but is mainly expressed in the CNS, especially in the hippocampus and cortex. BDNF is the most abundant neurotrophic factor in the body and acts by binding to TrkB, an intracellular region with intrinsic tyrosine kinase activity, which is activated by BDNF binding to TrkB, causing enhanced phosphorylation of TrkB itself, which in turn activates the Ras-MAPK pathway and finally activates CREB at the final activation of CREB at the serine site of CAMP response element binding protein (CREB). CREB promotes neuronal cell survival, increases synaptic plasticity and neurogenesis by increasing the expression of BDNF gene and anti-apoptotic protein gene BCL-2 (Lu et al., 2013). The mode of action is specifically as follows: (1) Increases synaptic plasticity, which in turn affects LTP, the process underlying the learning process and the process of memory formation (second level memory). (2) Promoting neurogenesis especially in the hippocampus, Ernfors et al. found that heterozygous mice with BDNF knockout had much less neuropathy than wild-type mice. (3) Promoting cell survival, mainly by maintaining and promoting the developmental differentiation and growth regeneration of various neurons, especially 5-hydroxytryptamine (5-HT) and dopaminergic (DA) neurons (Allen et al., 2013; Gao et al., 2022). BDNF not only binds to the Trk family of receptors (TrkA, TrkB, TrkC) but also to the P75 neurotrophin receptor, a member of the tumor necrosis factor family with a molecular weight of 75 kD, exert the corresponding biological effects. P75 receptors are not required for the function of neurotrophins (NTs); the role of P75 may lie in enhancing the affinity of Trk receptors for NTs, facilitating nerve terminal uptake and retrograde transport (Eggert et al., 2022). In addition, BDNF inhibits the pro-inflammatory signaling pathway NF-kB, thereby inhibiting the secretion of pro-inflammatory factors activated by glial cells and exerting neuroprotective effects (Cai et al., 2014). Some scholars believe that neuronal damage caused by decreased DNA repair capacity due to DNA oxidation is often observed in AD, and that BDNF can counteract DNA damage and increase transcription factor activity by increasing protein expression of purine-free/pyrimidine-free nucleic acid endonuclease/oxidation reductase-1 (APE1/Ref-1) through the BDNF/CREB pathway (Yang et al., 2014). For aberrant phosphorylation of Tau, the possible signaling pathway of BDNF action is PI3K/AKT. In addition to this, increased BDNF can induce a shift in APP degradation toward the α-secretase pathway and prevent the hydrolysis of APP to Aβ by β-secretase.

### 3.2 Muscle-exercise-exerkines

The peroxisome protein, FNDC5, is a glycosylated type I membrane protein. FNDC5 and its small molecule, irisin, produced by protein hydrolysis, play a considerable beneficial role in the course of AD. FNDC5 is widely distributed throughout the body but is upregulated in skeletal muscle, serum and hippocampus after exercise stimulation, probably because its upstream regulator PGC-1α expression is upregulated under exercise conditions (Wrann et al., 2013). FNDC5/irisin is a biological target that regulates AD and its expression level is negatively correlated with AD-related symptoms, such as improving neuroinflammation and promoting increased synaptogenesis and plasticity (Lourenco et al., 2019), Another ability of irisin is its permeability to the BBB, and irisin crosses the BBB to upregulate BDNF expression (Guo P. et al., 2022). In AD patients, induction of hippocampal proliferation is one of the potential therapeutic tools, and irisin can promote hippocampal and neuronal proliferation and improve synaptic function by upregulating BDNF expression and subsequently STAT3 signaling (Rabiee et al., 2020). After moderate treadmill exercise, improved spatial learning and memory abilities were observed in mice, possibly because exercise blocked the binding of Aβ42 to neurons via AMPK/PGC-1α/FNDC5/irisin (Azimi et al., 2018). In addition to these effects, it was also observed that increased expression of irisin inhibited astrocyte phosphorylation and IkBα inhibition due to Aβ exposure, thereby suppressing NF-kB activity and alleviating neuroinflammation. Irisin is known as the “messenger goddess”, and its beneficial effects on AD cannot be separated from the regulation of FNDC5 and BDNF, and more importantly, from their activation by exercise.

CTSB, a cysteine protease, is a muscle " Exerkines” that is thought to be induced in large amounts by exercise and mediates an increase in BDNF and DCX, thereby causing an improvement in hippocampal function (Suzuki, 2016). In a clinical study, 26 weeks of treadmill training was found to increase CTSB expression and show beneficial cognitive changes in middle-aged and older AD patients (Gaitan et al., 2021). However, at the same time, some scholars have taken an opposing view, suggesting that CTSB may increase the activity of NLRP3 inflammatory vesicles and induce oxidative stress (Bai et al., 2018).

IL-6, a factor currently thought to be induced by exercise, seems to play a “paradoxical” role in the organism. In healthy individuals, IL-6 expression levels were found to increase dramatically after exercise and return to normal levels during rest periods, and IL-6 is thought to inhibit the expression of the pro-inflammatory factor TNF-α (Ng et al., 2018). At the same time, however, chronic elevation of IL-6 is thought to be detrimental to the normal physiological function of the CNS, and large amounts of IL-6 are found in aged plaques in AD brains. In addition, IL-6 can also cross the BBB, whose stability is destabilized by AD, and transmit peripheral inflammatory processes into the CNS (Silva et al., 2021). Whether the specific role of IL-6 in the AD population is pro-inflammatory or anti-inflammatory needs to be discussed and explained by more scientific studies in the future.

### 3.3 Adipose tissue-exercise-exerkines

APN is an endogenous bioactive peptide or protein secreted by adipocytes that plays an effect on insulin sensitization. It has been found that APN is involved in and ameliorates some metabolic diseases that may induce AD, such as T2DM and insulin resistance. Exercise has considerable benefits in inducing improvements in the CNS involved in APN (Song and Lee, 2013). First, the effect of exercise on the induction of ADN is due to the activation of the AMPK signaling pathway stimulated by exercise. Activation of AMPK upregulates the expression of ADN, and the AdipoR1 (a receptor for ADN)/articulin, which is involved in the regulation of ADN, enhances hippocampal neurogenesis. The benefits of ADN for the hippocampus also include the promotion of its proliferation, relying on the AND/p38MAPK/GSK-3β/β-catenin axis to achieve this. In behavioral tests in mice, it was found that mice with normal ADN expression showed better cognitive function and LTP than knockout mice (Liu et al., 2020). As for neuroinflammation, perhaps two aspects can be discussed. A. Exercise induces the release of ADN, which in turn alleviates neuroinflammation via AdipoR1/AMPK/NF-kB/IL-1β. B. Acrp30/PPARγ, induces an anti-inflammatory M2 phenotype in microglia, secreting IL-4/IL-10, arignase1 and other anti-inflammatory factors. C. Mitigation of the disruption of BBB stability and permeability by neurotoxic substances of Aβ through Acrp30/AdipoR1/NF-kB axis. As previously described, the disruption of BBB has a facilitative effect on the development of neuroinflammation.

### 3.4 Liver-exercise-exerkines

IGF-1 is a tissue growth factor secreted by the liver and is found to be widely expressed in the nervous system, especially in the CNS, and it was found that animal studies in which IGF-1 signaling was blocked revealed exacerbation of some AD conditions, such as increased Aβ deposition and abnormal phosphorylation of tau, whereas in human and animal studies it was found that concomitant with increased physical activity, IGF-1 expression in the upregulation of IGF-1 expression in the CNS and the remission of AD disorders were found in human and animal studies, where the binding of IGF-1 to its receptor led to phosphorylation of its own protein kinase and activation of downstream signaling molecules, such as insulin receptor substrates 1 and 2 (IRS-1/2) (Ostrowski et al., 2016), which activated the phosphatidylinositol 3-kinase (PI3K)-Akt pathway. In addition, activated receptor tyrosine kinase aggregates the bridging protein Shc, which subsequently activates mitogen-activated protein kinase/extracellular signal-regulated kinase (ERK) 1/2 via a cascade reaction. In addition, IGF-1 is useful for neural stem cell proliferation, possibly through PI3K/AKT signaling to induce differentiation of neural stem cells (Trueba-Saiz et al., 2017). In addition, IGF-1 has a regulatory effect on APP metabolism; IGF-1 promotes the α metabolic pathway of APP and facilitates the transfer of β/γ metabolic pathway to α metabolic pathway, and also inhibits the expression of BACE1 thereby suppressing Aβ deposition (Jiang et al., 2017).

### 3.5 Exerkines in other tissues

Nerve growth factor (NGF) is the first identified neurotrophic factor that has the biological function of nourishing neurons and promoting protrusion growth, and has an important role in the development, differentiation, growth, and regeneration of central and peripheral neurons, and is widely expressed not only by neurons and glial cells, but also detected in peripheral cells, and reduces neuronal death in AD via the NGF/TrkA/PI3K pathway. A more consistent view is that in AD, NGF/TrkA mainly acts on non-amyloid proteins, and overexpression of NGF regulates TrkA binding to APP and promotes Golgi transport of APP, inhibits APP binding by BACE1, thus reducing Aβ production and eliminating imbalance, in addition to finding that NGF promotes APP transfer to the α-secretase pathway (Aloe et al., 2012). After exercise, NGF expression was increased, suggesting that exercise may have an important role in upregulating NGF expression.

In addition to NGF, another “Exerkines” widely distributed in various tissues and cells, VEGF, a highly specific pro-vascular endothelial cell growth factor, is also significantly regulated by exercise. VEGF has six structurally similar proteins, VEGF-A/B/C/D/E and placental growth factor. The VEGF family is known to promote increased vascular permeability, vascular endothelial cell migration, proliferation and angiogenesis (Melincovici et al., 2018). As mentioned above, changes in vascular function are often found in AD, which could also explain the decrease in VEGF expression levels found in AD. However, increasing physical activity in the course of AD upregulates VEGF expression, which is useful for hippocampal neurogenesis and improved synaptic plasticity, while the polymer of VEGF-C/VEGFR3 is useful for alleviating neuroinflammation, which not only induces M2-type polarization of microglia and promotes the production of anti-inflammatory factors, but also prevents neuronal apoptosis to some extent (Han et al., 2014). VEGF also appears to be reported to be associated with reduced Aβ deposition and improved abnormal phosphorylation of Tau proteins (Mahoney et al., 2021).

### 3.6 Enzymes

#### 3.6.1 Enzymes involved in the degradation of Aβ

NEP, an enkephalinase, is a type II membrane metalloendopeptidase with a zinc-binding motif (HEXXH) at the active site in the extracellular carboxy-terminal structural domain. NEP is highly expressed throughout the body, and in the CNS, NEP is found mainly at the presynaptic neuron terminals. NEP expression is upregulated by increased physical activity and also increases its enzymatic activity, an effect found mainly in the hippocampus and cerebral cortex (Hui, 2007). In AD, hemizygous NEP deficiency is often found, and it is hypothesized that NEP deficiency is associated with impaired memory, astrocyte activation and increased Aβ deposition. NEP acts as an Aβ degrading enzyme, mainly acting on BACE1, and NEP expression upregulates consistent BACE1 production, thereby inhibiting Aβ deposition production and age plaque formation (McAllister et al., 2010); NEP may also alleviate Aβ toxicity through BBB penetration.

In addition to NEP, there is an enzyme that specifically targets β-constituents. Insulin-degrading enzyme (IDE) is a metalloproteinase, and although insulin is the primary substrate for its action, its catabolic cleavage for other peptides gives it the effect of degrading Aβ deposition. One study found that increased physical activity significantly upregulated IDE expression levels in the murine hippocampus, and it was in the presence of AD that IDE concentration and activity were significantly reduced in patients. It is hypothesized that exercise may upregulate IDE expression through the PPARγ/AMPK pathway so that the accumulation of Aβ42 is reversed (Li et al., 2009; Costes and Butler, 2014).

#### 3.6.2 Antioxidant enzymes

Oxidative stress is an important mechanism of the aging process of the body and may be harmful for CNS (Salim, 2017). Both free radicals and reactive oxygen species (ROS) “contribute” to the process of AD, such as increasing damage to synaptic plasticity and triggering neuroinflammation, but small amounts of ROS are irrelevant to AD, mainly because of the presence of oxidative stress defense systems consisting of antioxidant enzymes that can scavenge them, such as superoxide dismutase (SOD) and catalase. However, the expression of antioxidant enzymes in the body is not constant, and when the level of ROS and other substances exceeds and suppresses the antioxidant enzymes, the oxidative stress state in the body surges. As two major categories of pathological mechanisms in AD, abnormal phosphorylation of Tau and deposition of Aβ contribute to the production of ROS. Aβ may cause ROS production by causing mitochondrial dysfunction and lipid peroxidation of unsaturated fatty acids. An important risk factor biomarker in the course of AD is RCAN1, and upregulation of RCAN1 is thought to result from oxidative stress caused by a combination of Tau phosphorylation and Aβ (Greenough et al., 2013; Chen and Zhong, 2014; Kim et al., 2015).

SOD is a metalloenzyme whose defense against oxidative stress depends mainly on the scavenging of superoxide anion (O2-). There are three types of SODs, distinguished by the different metal ions they contain: SOD1 contains Cu2+ and Zn2+; SOD2 is an SOD containing Mn2+ mainly in mitochondria, and SOD3 is an SOD containing Cu2+ and the only secretory SOD. Physical exercise significantly increased SOD1 and SOD2 in the *in vivo* study. In AD mice, the Aβ42/Aβ40 ratio decreased with increased SOD2 and restored LTP (Ma et al., 2011). Overexpression of SOD1 prevents superoxide anion and oxidative stress induced by polymers of Cu and Aβ (Murakami et al., 2012).

Glutathione (GSH) is a gamma-amide-bonded and sulfhydryl-containing tripeptide composed of glutamate, cysteamine and glycine and is present in almost every cell of the body. It is commonly believed that oxidative stress-mediated AD is associated with reduced GSH expression. Studies in humans and animal models have shown that physical exercise is effective in increasing GSH content and GSH/GSH persulfide ratio in the CNS. GSH protects brain endothelial cells by inhibiting NO and ROS production, and in addition, upregulation of GSH expression activates the activity of the Nrf2 signaling pathway, and many downstream target factors under its control are often considered to be key antioxidants. GSH also has the potential to deplete and induce activation of pro-inflammatory signaling pathways in microglia and astrocytes, leading to the release of pro-inflammatory factors and exacerbating neuroinflammation. Similarly, for Aβ, a major pathological marker of AD, GSH deficiency and reduction contribute to Aβ formation, probably because GSH deficiency causes the accumulation of carboxy-terminal fragments of Aβ precursor proteins (Saharan and Mandal, 2014; Braidy et al., 2015; Mandal et al., 2019; Lin and Lane, 2021).

## 4 Discussion

The issue of population aging is something that not only developed countries need to face, but also developing countries should consider. The changes in social life have amplified the harm of AD for individual members of society and the whole social aspect. The exact cause of AD is still unclear, and the treatment is relatively single, so how to prevent and treat it is a problem that every concerned person should consider. As an inexpensive and widely beneficial tool, the benefits of physical activity are cross-disease and cross-age, and increasing regular physical activity seems to be more effective for those with both AD and other diseases than the specificity of the effects of medications used to treat AD, which is true, as AD patients are often found to have other chronic diseases, and the greater benefit of physical activity is not curative but preventive (Suzuki, 2019).

This review takes three classical AD-related signaling pathways as the starting point and adds the perspective of “Exerkines” to summarize and discuss the current representative studies to reveal the molecular mechanisms of exercise to alleviate AD-related disorders. In short, exercise itself “Exerkines” released by motor stimulation act on three classical signaling pathways, AMPK, Wnt, and PI3K/AKT, or directly at the site of the disease to alleviate neuroinflammation, improve synaptic plasticity, promote neurogenesis, reduce Aβ deposition, decrease abnormal tau protein phosphorylation, maintain BBB stability, and improve autophagy, thereby reversing the effects of neuroinflammation, reduced synaptic plasticity, neuronal apoptosis, decreased memory capacity, and degeneration of higher cognitive abilities caused by AD.

Although exercise has been observed to be effective in the prevention and relief of AD in some human studies, and the same findings were reported in a summary meta-analysis (Farina et al., 2014; Hernández et al., 2015; Kivipelto et al., 2018), we believe that these studies are more focused on the discovery of “macroscopic phenomena”, although the rehabilitation of AD patients through exercise or the reduction of the risk of AD in healthy people through exercise is our ultimate goal. Although rehabilitating AD patients with exercise or reducing the risk of AD in healthy people with exercise is the ultimate goal, it seems that the complex molecular mechanisms of the interaction between AD and exercise need to be more finely “micro-explained” so that readers or researchers can consider the effect of exercise on AD prevention and treatment through both micro- and macro-validation when viewing the relevant articles. This is necessary for a scientific and critical study of the field of “exercise intervention in AD”. Therefore, although evidence from human studies is collected, the focus of this paper is more on the complex interactions between AD and exercise *in vivo* through molecular signaling pathways.

While it is true that some high quality reviews have focused on these issues, our study differs from previous studies in that it breaks down several topical conditions related to AD that currently exist, and under each topical condition, describes the signaling pathways through which exercise may play a beneficial role in AD, which is more clearly organized and may be easier for the reader to read.

In addition, we have systematically described “locomotor factors”, substances regulated by locomotion, which are secreted under the conditions of locomotion, and which have rarely been covered and discussed in previous reviews.

In some studies it is considered that AD is one of the concomitant causes of increased sarcopenia and therefore muscle mass in AD patients is one of the concerns (Yun et al., 2021), and it is precisely in this regard that the benefits of regular exercise are also highlighted, and after training, whether aerobic, resistance or mixed, AD patients get beneficial results both in terms of muscle genesis and muscle mass, improving their daily life capacity. In addition to this, the researchers suggest that there may be a link between the decrease in muscle mass and hippocampal genesis, and that there is a positive relationship between muscle mass and cognitive performance (Lee K. et al., 2019; Moon et al., 2019).

Interestingly, in summarizing the above literature, we found that the effect of exercise on stimulating signaling pathways to inhibit AD may also involve a cascade of responses between different signaling pathways, rather than a simple “one-way” unilinear development. In addition, many natural extracts of plants belonging to polyphenols may also have a mitigating effect on AD, such as epigallocatechin gallate, curcumin, resveratrol, *etc.* Moreover, the ameliorating effect of these substances on AD may also be produced through the stimulation of some of the above-mentioned signaling pathways, which is similar to the mechanism of the ameliorating effect of exercise on AD. Therefore, we hypothesize that the combination of polyphenol supplementation with increased physical activity may be a good approach, but unfortunately, there are few studies exploring the combined effect of exercise or polyphenols on AD compared to the effect of exercise or polyphenols on AD alone.

Although some studies have examined the effect of exercise on AD through the Wnt signaling pathway, there is a paucity of studies compared to “Wnt and AD” or “exercise stimulation of Wnt for other diseases”, and although this review also summarizes the current evidence based on exercise stimulation of Wnt to improve AD, there are still some elements that deserve further investigation by researchers to fill the gap.

Finally, recent research suggests that there may be an interaction between gut flora and the brain via the “brain-gut axis”, which appears to point to a new aspect of AD research, with the possibility that gut flora may be a new target for the treatment of AD. In summary, potential directions that still need to be developed in the field of exercise to improve AD include: A. Polyphenols combined with exercise for AD. B. Exercise through the brain-gut axis for AD. C. Exercise through the Wnt signaling pathway for AD.
